# The Experience of Social Exclusion and the Path to Inclusion from the Perspectives of Immigrant and Refugee Women in the Niagara Region

**DOI:** 10.3390/ijerph22010012

**Published:** 2024-12-25

**Authors:** Joanne Crawford, Tara Lundy, Chiarina Crawford, Jane Moore, Nicole Viscek, Nyarayi Kapisavanhu

**Affiliations:** 1 Department of Nursing, Faculty of Applied Health Sciences, Brock University, St. Catharines, ON L2S 3A1, Canada; tl16kj@brocku.ca (T.L.); jmoore4@brocku.ca (J.M.); 2 Altum Health, Stoney Creek, ON L8G 1B5, Canada; chiarina.crawford@uhn.ca; 3 Tools of Empowerment for Success (TOES Niagara), Welland, ON L3B 3W7, Canada; nicole@toesniagara.ca (N.V.); nyarai@toesniagara.ca (N.K.)

**Keywords:** immigrant, refugee, women, social exclusion, social inclusion, migration, strategies, settlement, integration, community

## Abstract

Social inclusion is a common goal for equitable access to resources for living, is important to health and wellbeing, and is supported by most Western or developed nations. Despite this, immigrant and refugee women continue to be excluded from social, cultural, economic, civic, and political participation during and after settlement. Most research exploring the context of social exclusion has reinforced that some groups experience greater exclusion than others in any given population, for example, immigrant women. The purpose of this study was to gain insights by exploring the experiences of social inclusion and exclusion and recommendations from the perspectives of immigrant and refugee women, as well as community service workers in the Niagara Region, Canada. Utilizing qualitative descriptive inquiry underpinned by intersectionality theory along with thematic analysis, we interviewed 10 immigrant and refugee women and 14 community service workers. Five themes were generated: (1) gendered nature of exclusion; (2) levels of exclusion; (3) paving a path for self; (4) formal inclusionary processes; and (5) informal inclusionary processes. The findings will be used to guide community action and may be transferable to community organizations that serve immigrant and refugee women in similar community contexts, with the aim of enhancing collaborations to advance health equity and inclusion.

## 1. Introduction

The mobility of migrants continues to increase in developed nations, with most choosing to migrate for work, family, or study or being pushed to leave their origin country because of conflict, persecution, and disaster, while smaller and more vulnerable cohorts are displaced, such as refugees or internally displaced persons [1]. Across the globe, numerous Organization for Economic Cooperation and Development (OECD) and European Union (EU) countries have adopted social inclusion policies to facilitate integration and settlement [2]. These are ranked based on performance, and while some OECD/EU countries’ policies are successful in facilitating social inclusion and equal opportunities, others lag behind, and such policies fail to prevent social exclusion or equity [2]. The social policies in existence include income transfers, such as child benefits and pensions, along with educational policies that serve to promote equal opportunities and social inclusion in Canada. In Norway, social policies incorporate family child allowances, paid-leave, and childcare subsidies, along with other social insurance benefits [2]. While Canada fairs well in the scoring, some groups, such as newcomers, continue to experience social exclusion [2]. Women made up 48.1% of global migrants in 2020 [1] and are more vulnerable to social exclusion [3]. Social exclusion is a multidimensional concept that is a state and process and has many definitions but is bound by a common feature, which is the lack of participation in society [4]. Individuals and groups, such as newcomer women, are not able to or are not given the opportunities to fully participate socially, economically, culturally, or politically in the sociocultural context. Immigrant and refugee women may experience social exclusion in new settlement communities due to multiple disadvantages, which make them feel that they do not belong; for example, they may lack social support networks, encounter barriers to labor market integration, and have low income [4]. Additionally, social exclusion is sustained by systemic processes that continue to exclude some groups over others despite the social policies that are in place, as these alone are not enough to promote inclusive societies. As a social determinant of health, social exclusion intersects with other determinants, including immigrant and refugee women’s social identities and structural processes that create social inequalities [3]. The level of social exclusion is dependent on the group, context, and time, and if people are subjected to material and social deprivation, this leads to social exclusion and affects their health and well-being [3]. The impact of social exclusion creates the social and living conditions and experiences that impact health. Marginalization and social exclusion are the main issues that lead to a host of chronic diseases, for example, depression and adult-onset diabetes [4].

Across the literature, immigrant and refugee women have been more vulnerable to social exclusion in Canada, the United States of America (USA), the EU, the United Kingdon (UK), Australia, and New Zealand [5,6,7]. The migration experience, including norms, expectations, and power relations, serves to shape immigrant and refugee women’s experiences of vulnerability and oppression that lead to exclusion [7]. A critical review of the literature provided insights into the causes of social exclusion and inclusionary processes that support inclusion for immigrant and refugee women settling in Western contexts [5]. While the review findings focused on social exclusion and inclusion, often, this was in the context of acculturation, health promotion, or other needs and indirectly linked to social inclusion and exclusion upon settlement. Therefore, this study addresses a gap, as patterns of settlement differ across contexts, and to fully understanding immigrant and refugee women’s experiences, specifically social exclusion and inclusion, it was necessary to seek out experiences in the local context of the Niagara Region.

### 1.1. Purpose and Objectives

The purpose of this study was to gain depth of understanding and insights into the experiences of social inclusion and exclusion and recommendations to promote inclusion from the perspectives of immigrant and refugee women, as well as community service workers in the Niagara Region, Canada. The study objectives include the following: (1) to explore the experiences of social exclusion and inclusion and strategies that are culturally relevant and appropriate to promote inclusion, as perceived by immigrant and refugee women in the Niagara Region; (2) to identify the structural processes of social exclusion and programs and services that incorporate social inclusion strategies from the perspectives of community service workers who serve immigrant communities; and (3) to develop the evidence to inform tailored social inclusion strategies for immigrant women in our community but also contribute by advancing knowledge that may be used by others in similar communities and contexts.

### 1.2. Literature Review

In the following text, we report on findings from our critical review of the literature that explored the causes and outcomes of social exclusion among immigrant and refugee women settling in Western contexts [5], which was conducted as part of a larger three-phase project in the Niagara Region, Canada.

To explore the experiences of social exclusion, a critical review of the pre-existing literature identified 60 scholarly papers and reports, of which the majority were inquiries that explored immigrant and refugee women’s perspectives using qualitative inquiry; 45% were studies undertaken in the Canadian context [5]. At the individual level in the social environment, isolation and loneliness were common themes across studies that focused on both younger and older immigrant and refugee women. Among younger women settling in the EU, the UK, Australia, and Canada, the challenges with the time allotted for integration and roles and responsibilities such as caregiving [8,9,10,11], as well as being a single parent with limited language skills and social and financial security [12], made women feel isolated and excluded. The cultural transition was difficult for immigrant and refugee women in the USA, Sweden, Australia, and Canada due to the adjustment to the new cultural context, for example, societal values, social structures, and the host language [13,14,15,16].

Isolation may lead to loneliness, and this transcends time; some immigrant women with origins from Hong Kong that settled in Canada were interviewed eight years after migration, and the loss of friends who returned to their home country along with lack of desire and confidence conversing in English with outsiders contributed to loneliness and, in turn, the desire to integrate [17]. Likewise, older South Asian women settled in Australia, Sweden, and Canada felt isolated because of limited social relationships given the loss of social supports upon settlement and limited to no opportunities to develop relationships [10,14,18,19,20,21].

A woman’s social support network is integral to the relationships cultivated upon settlement and thereafter but is also relevant in the context of the meaning of these supports and feelings of exclusion. The loss of social support following migration, lack of friends or informal supports, meaningful and satisfying relationships, or opportunities to socialize with others were common themes described by immigrant women [10,11,14,18,22]. In Canada, often the main support for Korean women was a partner or spouse [23] or family member for older South Asian women [24]. For older Chinese widows, the lack of familial or friend support was related to children being busy with work and grandchildren, friends passing away, and weakened transnational supports [25], and for single immigrant mothers living in Sweden, family living at a considerable distance limited social supports [9]. Reliance on family not only provides social support but instrumental support to navigate the language and social spaces.

A lack of proficiency in the language of a settlement country was an important factor that excluded immigrant and refugee women from participation in social, economic, cultural, and civic activities. Immigrant and refugee women who experienced language barriers were unable to engage with the host culture, access social supports, navigate spaces to obtain resources for living, secure employment, or have opportunities to engage in intercultural conversations with the host society [9,12,13,14,18,19,22,23,24,25,26,27,28,29,30,31,32]. Over-reliance on family for language support or feeling uncomfortable to negotiate social spaces or transit also served to exclude some women in Canada and Australia [23,25,28,32,33].

At the community level, immigrant and refugee women experienced greater exclusion due to (1) the loss of social status upon settlement [18,19,22,23]; (2) the loss of independence, their career, and professional identity [34]; (3) the loss of identity and some traditional values [19]; (4) the loss of individual autonomy [8]; and (5) racism and overt discrimination [16,35]. In Canada, immigrant women experienced interpersonal discrimination in the labor market related to undervaluing their prior education or professional credentials and poor renumeration [21], had less options for career opportunities [30], or they were exploited, which contributed to social exclusion and lowered social status [12]. For women who entered the labor market to supplement household income or as the main income earner, they were faced with unfamiliar, low skilled, or precarious working conditions [6,23]. For some, caregiver and household responsibilities were challenging for immigrant women to obtain employment [9,16], and sometimes, a lack of spousal support resulted in women needing to withdraw from career training [9,23]. The unfair or unjust assessment of the educational backgrounds or professional training of immigrant women was exclusionary [26,29,33].

Across larger systems, exclusion from access to social and health services was reported; for example, some immigrant and refugee women felt that there were limited culturally sensitive services to enable access, such as interpreters [14]; limited knowledge and awareness of what they could access because of the confusing and complicated systems [15,16]; and limited options for financial support from government programs [16].

The causes and outcomes of exclusion were evident from the literature, as were the processes of inclusion, which included micro-, meso-, and macro-level processes [5]. For example, the necessity to support immigrant and refugee women to build social support networks including both bonding and bridging social capital, creating opportunities to be engaged in communities through socializing or volunteer activities, and government/institutional programs that tended to the sociocultural needs of women during and long after settlement. The critical review led to the development of an initial conceptual model underpinned by an intersectionality framework for immigrant and refugee women [5] and served to guide the current qualitative study.

A critical and intersectionality lens allows us to gain a deeper understanding of the social locations and diverse experiences in relation to the social identities of immigrant and refugee women that not only addresses gender but other interesting identities of age, race, class, ethnicity, nation, and ability, to name a few [36,37]. The intersectionality lens was applied throughout the research process to shed light on social identities focusing on the sociocultural context to elucidate systems of power relations that have historically oppressed and created social inequalities among immigrant and refugee women during and after settlement in Western societies. Adaptation requires the consideration of prior experiences, social identities, as well as the re-establishment of identities within a society that has different systems and views on gender and the roles of women in a family. Therefore, this study explores the experiences of social exclusion among immigrant and refugee women through a critical intersectionality lens to inform settlement, multicultural organizations, and local or national policies on actions to promote social inclusion in communities of settlement.

## 2. Materials and Methods

The Social Justice Research Institute at Brock University works closely with communities, and this project came from a fall symposium where community organizations were able to share community issues. Tools of Empowerment for Success, Niagara (TOES Niagara), a not-for-profit community organization that provides community-based supports, brought forward a local issue of social exclusion experienced by immigrant and refugee women attending the center. A long-standing partnership and collaboration were formed to work together, and this project is a culmination of a larger community-based research project.

A qualitative descriptive design was employed using two semi-structured and open-ended interview guides framed within an intersectionality lens [36,37]. Qualitative descriptive methodology aligns best with the research objectives, as it entailed the gathering of rich data from the perspectives of immigrant and refugee women, as well as community service workers who serve newcomer women in the community [38]. Staying close to the participants’ words is important to gain diverse and multiple subjective experiences that aim to inform recommendations for settlement and community services. We used intersectionality theory as a guiding framework during data analysis [36,37]. The study was undertaken from June 2021–January 2022 in the Niagara Region in Ontario, Canada. Research ethics approval was obtained from the Health Sciences Research Ethics Board from the Office of Research Ethics, Brock University.

### 2.1. Recruitment Setting

The Niagara Region, a mid-sized, second-tier regional center with 12 municipalities, represents a mix of rural, suburban, and urban communities, and the organization of social and health services is complex. Over the last number of years, the region has been welcoming immigrants and refugees in increasing numbers; however, these communities experience challenges and exclusion.

At the time of the study, we were functioning within a global pandemic. Therefore, in keeping with public health restrictions, our team disseminated electronic posters to multicultural centers in the region and to the local immigration partnership to reach immigrant women across all communities, as well as community service workers that support immigrant and refugee women in the Niagara Region. Additionally, the dissemination of news releases through Brock University to community partners took place at two different time points during the seven months of recruitment.

### 2.2. Sampling and Participants

In collaboration with TOES Niagara, the recruitment of immigrant and refugee women and community service workers occurred simultaneously. Purposive sampling was used following the established inclusion and exclusion criteria to recruit immigrant and refugee women from diverse communities; snowball sampling was used to expand our reach, particularly at a time when restrictions made it challenging due to a lack of on-the-ground recruitment. Participants who self-identified as adult immigrant and refugee women who were residents in the Niagara Region for any length of time were eligible for the study. Temporary student residents were excluded. For community service workers, those who were mandated to provide services to newcomer, refugee, and immigrant women (or men) and those who provided informal or formal services or programs that were targeted to social, cultural, economic, or political aspects of social inclusion in Niagara were eligible to participate. Individuals that provided temporary resident services were not eligible.

### 2.3. Procedures: Immigrant and Refugee Women

Posters disseminated through multicultural centers that women accessed included email and telephone contact information to reach out if they were interested to volunteer for the study. The participants contacted the research assistant, who then confirmed their eligibility, provided further information on the study and purpose, emailed an information sheet and consent form, and booked a 45–60 min virtual interview at a convenient time depending on the participants’ availability. Once eligibility and informed consent were established, the participant provided verbal consent and received an online link to a sociodemographic form to complete. Interviews were audio-recorded with expressed permission, and summary notes were taken to allow for member checking at the end of each interview, which was feasible to the context and population.

### 2.4. Procedures: Community Service Workers

A letter of invitation was disseminated through immigrant and settlement organization contacts, and if community service workers were interested in volunteering, they were directed to a website to complete a brief survey questionnaire. At the end of the survey questionnaire, they were asked if they wished to participate in an individual semi-structured interview. If they left a contact email, one research assistant emailed the participant to set up a time for the interview, provided informed consent, and held a virtual interview using Microsoft Teams or a telephone interview. The interviews ranged from 30 to 45 min with audiotaping with participant consent. Summary notes allowed for member checking at the end of the interview.

### 2.5. Data Collection

The data collection processes differed for each group of participants only with respect to the interview guides utilized. Our approach in interviews was the same for each group, as we wished to create a safe space, equalize power differentials, and develop rapport so immigrant and refugee women and community service workers (all women) would feel comfortable to share their experiences. Two research assistants conducted the interviews. The individual interviews encouraged immigrant and refugee women participants to share their individual perspectives, with probing for clarification, and provided an opportunity for greater depth of exploration of their unique cultural and social experiences. Additionally, the individual interviews accommodated the needs of community service workers recruited from community organizations to share their experiences working in the community with diverse newcomer women. Two semi-structured interview guides were developed, tailored to each group: one for immigrant and refugee women and one for community service workers. The interview guides were pilot tested with the first two participants in each group of participants. The questions for immigrant or refugee women focused on sharing experiences of feeling socially isolated or excluded in their community: for example, “what things make it hard for you to participate from a social or cultural perspective? Among the questions for CSWs, we asked about the women they worked with and collaborations with other organizations to promote inclusion: for example, “What process need to be in place to enable access and social inclusion for immigrant women in your community? The data collected were transcribed verbatim using Microsoft Teams software and checked for clarity and completeness by the research assistant who did not conduct the interview. The data were stored in a password-protected shared drive to be accessible and accommodate data analysis with multiple partners on the team.

### 2.6. Data Analysis

Transcripts of the data were analyzed using thematic analyses guided by intersectionality theory [36,37] and Braun and Clarke’s [39] five phases of inductive analysis of qualitative data, with four team members being responsible for analyzing data. The investigators began by individually familiarizing themselves with the data, which involved reading and re-reading the textual data from transcripts of the interviews. During this process, two team members were partnered and responsible for individually reading the same set of transcripts to make casual and observational notes and generate initial codes that closely reflected the data and meaning ascribed by the participant quotes for phase two. A team meeting was held to discuss individual coding to capture the diversity of the patterns within the data and relate them back to the research objectives. In phase three, another team meeting was held to assess the overlapping patterns among codes to generate common themes that meaningfully represented the data. A final team meeting was held for the fourth phase to review and refine the themes to reflect the most relevant aspects of the meaning of the experiences of immigrant and refugee women and community service workers. In the final phase, the themes were finalized and defined.

### 2.7. Rigor and Trustworthiness

We employed several measures to enhance rigor. The creation and pilot testing of the semi-structured interview guides was facilitated with two interviews with both groups—immigrant women and community service workers—and this allowed us to refine the guides for comprehension, make additions, and add probes. Audio recording and verbatim transcription along with summary notes were important to ensure we captured the experiences, which contributed to the authenticity of the findings. The collaborative and systematic process of coding during thematic analysis and reflexive journaling added credibility to the study. Debriefing among team members and member checking after the interviews enhanced the credibility, critical interpretation, and trustworthiness. The lead researcher kept an audit trail, and the entire team engaged in ongoing reflexivity.

### 2.8. Reflexivity and Positionality

Each team member engaged in reflexive journaling to critically self-reflect, appraise, and evaluate their subjectivity and social context continuously throughout the research process. Collaborative sharing of assumptions and reflections allowed us to keep true to participants’ experiences. The lead researcher, also a White immigrant woman, had extensive public health experience working with diverse immigrant women in several regions. This experience enriched the understanding of social and health system challenges that immigrant and refugee women face daily. All team members reflected on positionality. During data collection, we aimed to reduce power differentials and create safe and comfortable spaces for immigrant women to share their experiences. Reflexivity and positionality were integral to data analysis and dissemination activities.

## 3. Findings

A total of 10 immigrant and refugee women (IWs) and 14 community service workers (CSWs) were interviewed. Overall, the IWs originated from Colombia, the United States of America, Jamaica, Sudan, Mexico, Burma, and Zimbabwe. The age of IWs ranged from 20 to 67 years, residency in Canada ranged from 3 to 39 years, and most participants were employed more than 30 h a week (Table 1). CSWs were employed in various communities across the Niagara Region based on the population served, programs offered, outreach methods, and partnerships (Table 2). Sixty-nine percent of CSWs reported that they did not have a program whose primary goal was to promote social inclusion, and of these, all indicated that there was a greater need to promote social inclusion for IWs.

The experiences of social inclusion and exclusion among IWs and CSWs in the Niagara Region provided insights and enhanced understanding of gendered identities that created obstacles for women to participate in social, economic, cultural or civic activities; levels of exclusion that were linked to intersections of social identities; structural level restrictions; experiences of making connections; and subsequent increased confidence and empowerment. Participant perspectives reflected individual feelings, thoughts, and context within which women felt socially excluded in their new environments, as well as the positive aspects of social inclusion in places and spaces within their new community. Distinctions between the perspectives of IWs and CSWs will be provided to give voice to each perspective in the following presentation of themes and sub-themes.

### 3.1. Theme 1: Gendered Identities

Throughout the interviews, IWs discussed their gender roles, loss of identity and status, expectations of migration, and unique experiences of their ways of being. Gendered identities encompassed the unique experiences of women, including ways in which IWs navigated roles, sought out settlement services, and how this impacted them. Two sub-themes of pressures and priorities as well as isolation and not belonging will be presented.

#### 3.1.1. Sub-Theme: Pressures and Priorities

IWs reported finding it difficult to juggle the pressures that come with settlement but also discussed prioritizing the needs of their family, including the emotional work in the family, caregiving needs, and putting their husband first so that they may actualize their career aspirations. One CSW, also an immigrant woman, highlighted the gendered nature of her struggles but also reinforced the cultural role of women within her family unit:

*… I have two kids, I have to learn the language, and I have to find a job so it’s all the pressure on people. Sometimes when you go to the multicultural centers you got to study, you have to learn, you have to find a job … I felt [pressure] because as women we need more support … sometimes it’s more difficult for us to reach our professional goals when we are here [in Canada] because we are the ones who stay home with the children …*

(CSW01)

Prioritizing family and partners careers were common among IWs accessing a multicultural center in Niagara, which delayed their entry into the labor market.

*I see it in a lot of women too and I have clients that come in and they are highly educated in their country, like they’re doctors, they are lawyers …, they feel like they come here and all they want to do is look after the family because the husband has to succeed before they can.*

(CSW07)

Some IWs struggled to balance multiple social identities, such as being a caregiver and the primary income earner in the family household, while trying to acculturate as a contributing member into a new society. For instance, one refugee woman participant (IW10), felt “frustrated” when trying to make ends meet as a daughter who was managing her father’s depression, saving money for school, and being the sole income earner in her household.

*I feel like I don’t belong, that’s the truth. I feel like I don’t belong in this community because it seems like nobody understands what I’m going through. Nobody understands my pain. For example … I will go and say like I need hours because this is happening to my family and then they act like they understand and still not give me the hours.*

(IW10)

#### 3.1.2. Sub-Theme: Isolation and Not Belonging

Some IWs described feeling isolated in different ways, and for the most part, this was experienced in diverse social contexts. A lack of diversity and representation of women from their culture in social settings made it harder for the women to feel that they belonged, and this impacted their health.

*When I first arrived in Canada, I felt isolated because I moved to a new place and I didn’t know a lot of people in the area and it was my first time to be away from family … so I really felt isolated … I went into depression … I was thinking maybe I made the wrong decision moving to Canada, because you know, I was alone.*

(IW11)

Another participant described cultural transitions, a sense of isolation, and exclusion upon settlement in the community.

*The biggest time in my life that I felt isolated was when I first arrived in Canada. It was just a huge culture shock … all the things I grew up knowing … it was like a huge turn that things were just different here … there was not a lot of Black people where I lived and there was not a lot of Black people in my school. People treated me as a novelty, and so I just kind of felt isolated because of that, because I was the only one, like me.*

(IW08)

Even with many years of settlement in Canada, IWs felt like they did not belong because of subtle acts of exclusion in workplaces or other settings. IW13 expressed how she felt “*every time* [she] *opens* [her] *mouth,* [she] *becomes a newcomer … you’re never going to not be an immigrant*.” While something may seem trivial and normalized, it continues to exist in the background to make women feel excluded.

*So, in my case, I’ve been here 20 years, it’s more subtle day activities that you don’t feel like you belong. Not so much big racism that I used to see when I came when I immigrated 20 years ago.*

(IW13)

Being attuned to the intersecting social identities of IWs participants, considering cultural values, multiple roles, and pressures upon settlement, and prioritizing family and partners are important aspects that shed light on factors that may impact exclusion. Moreover, experiences of feeling isolated and not belonging because of a lack of exposure to diverse populations in community spaces contributed to the social exclusion experience.

### 3.2. Theme 2: Levels of Social Exclusion

The ways in which exclusion was portrayed by participants reflected interactions with the host country at different levels: interpersonal, community, and system. Unique differences in culture and prior experiences as well as limited diversity in social spaces contributed to this feeling of exclusion. The sub-themes discussed in the following section include the intersectionality of oppression, othering and everyday racism, inclusion with restrictions, and struggles with transitions.

#### 3.2.1. Sub-Theme: “Intersectionality of Oppression” Determines Exclusion

CSWs witnessed diverse levels of oppression; the more challenges women had (e.g., economically or socially), the less likely they were to participate. This was often seen among IWs who needed more time and effort from a service perspective as CSWs attempted to meet their needs.

*The pathway gets more and more complicated and difficult the more intersectionality of oppression somebody encounters … some of our most complex needs end up with older women … older women, who are persons of color, who you know are still suffering from decades of poverty and …, layers of oppression … we tend to spend roughly about 80% of our resources on the 20% of the most highest needs, women or individuals, and rightfully so we should and we ought to, because we know that the path for them is so much more complex than for all of us.*

(CSW17)

IWs residing in Canada for longer also faced social, economic, and cultural challenges and never felt they belonged. The emotional toll of exclusion persisted over time, and this resulted in feelings of frustration, resentment, and depression. IW14 (who declined audio recording) stated that she was “*heartbroken*” from her experiences of racism and discrimination over the course of her career trajectory, the lack of employment opportunities, and her treatment as an educator. In turn, IW14 felt this influenced her significantly because her career was an important aspect of her social identity that remained unfulfilled even after many years in Canada.

#### 3.2.2. Sub-Theme: Othering and Everyday Racism

Women participants felt othered by experiences through everyday acts of racism in social settings, and, in turn, this impacted their well-being and made them feel unwelcomed and not accepted. Some IWs felt that they received differential treatment and prejudice based on their race and ethnicity, while others shared that they felt they were avoided, ignored, and distrusted by others. Some IWs experienced name calling, racial slurs, and hurtful comments during community activities that contributed to feeling excluded. For example, IW10 described the experience of daily microaggressions and derogatory terms towards her, which made her feel “*depressed*” and “*sad because no one can relate*”.

*I had someone say like, “oh you look like Aunt Jemima” you know? Or just make comments about my hair or your hair is too dry, your hair looks dead, it’s looking dry.*

(IW10)

One IW participant enrolled in continuing education classes felt that she was perceived by peers as not being able to be as academically successful as others. These behaviors made her feel an internalized sense of “pain”.

*You know, I was so discouraged. I felt so sad. I felt belittled because like I was looking at myself thinking that OK because I’m Black people look down upon me …, I would get higher marks and people would comment. They were like surprised why I got higher marks than them. And, because I was like surprised because I’m Black I’m not supposed to be smart? Is that what they think?*

(IW11)

Another IW described her experience in an educational program where she was placed in a housing situation with a roommate but eventually was forced to leave due to bullying and living in fear on a daily basis. Past traumatic experiences resurfaced and made it untenable to stay in this environment.

*… I’m coming from work and I’m, you know, as you’re getting closer to the house, your heart starts racing and you’re thinking oh my goodness, I have to face that? What is she going to do today? When you start facing some things like that [bullying and fear], it kind of takes you back to those difficult times and then you start feeling those things you have been trying to get past you know … Like the trauma I face, yes, because you know, as an immigrant, I came here as a refugee, the trauma I went through … Yeah, so you are trying to get those things behind you and then now you have to deal with this every day and it evokes those emotions that you’ve been trying to like put behind you and get over …*

(IW15)

#### 3.2.3. Sub-Theme: Inclusion with Restrictions

While services for settlement and non-profit community organizations supported integration and provided invaluable services and opportunities for most, at times, eligibility for some programs was restricted, and this created exclusion.

*They send you to a program to help with you know, resume writing, cover letter writing, job search. It was a program for everybody, specifically Canadians, so the assumption is that you are born and raised here. There are certain terminologies that we [immigrants] are not used to. Nobody took that extra step to kind of explain and if I may say this, the services were not culturally sensitive.*

(IW09)

Supporting IWs was also problematic for CSWs, as the systemic processes may be exclusionary, for example, to obtain the required documentation to receive credit for past education and credentials or navigating the lengthy immigration processes.

*So, we are definitely advocating to do something where people can demonstrate their skills without having to go through this lengthy process of even trying to get an academic transcript sent directly from a university in a war-torn country or where there’s political unrest, during a pandemic. You know, like it can be really hard for them to be able to access what’s needed under the current system.*

(CSW06)

Government programs may facilitate the integration of newcomers; however, these are restricted to career, language, and employment domains and are less about social integration within the community. Aspects of community belonging and welcoming immigrants is left to informal programs.

*… sadly the government isn’t seeing as much value as they used to in conversation circles and stuff* [informal]*. And you know, I’m going to take that back. They see value in it. They’re just not choosing to fund it. Um, because they do believe in many respects that it should be a community-led response. It should not have to fall on the government to necessarily fund a community welcoming new community members.*

(CSW08)

Other barriers to employment among IWs were reported. IW09 shared that she needed to “*start all over*” in her career when she came to Canada because even though she held a graduate degree in her origin country, her professional credentials were not recognized. She shared her struggles to financially support herself while completing education in Canada; she accepted any job position that she called a “*survival job*” (IW09), which often involved undesirable work conditions, unstable part-time positions, night shifts, manual labor, and insufficient financial compensation.

#### 3.2.4. Sub-Theme: Struggles with Cultural Transitions

IWs faced other challenges related to instrumental support and social support, for example, securing housing and financial support, the inability to participate in social activities, and the loss of or no social support.

*It was very hard, it was challenging for us, trying to find a good place to live, we weren’t asking for much, but at least clean, right? Clean and affordable because the money wasn’t, like it was helpful, like I have to say we were thankful for it, because nobody offered you money just to be here, but it had to be, like we had to with a certain amount of money, we have to cover housing, food, transportation, and it wasn’t very much.*

(IW02)

The inability to provide a credit score as proof of financial stability was a challenge faced by some IWs in the process of securing housing in Canada upon arrival, despite government support. A lack of knowledge or limited ability to access community resources also made IWs feel excluded. In particular, transportation was described as an obstacle to participation and community integration. Many IWs relied on public transit as their only mode of transportation because they did not have a Canadian driver’s license, or they did not own a car; they experienced challenges due to limited transit services between communities. If it was difficult to access social spaces, then the IWs chose not to participate any longer, and this was the case for one participant who shared:

*We can never make it on time, because obviously we’re taking the bus and then coming back home like it’s a lot, especially when it’s cold. Sometimes you get stuck, you know the bus is late or the bus is not coming at all. We just, just stopped going. We stopped going [to church] because of that.*

(IW10)

Some IWs had a difficult time when first transitioning to their new home due to layers of cultural differences. For one woman who came to Canada in her teens, even the diversity of contexts made it difficult to connect or relate to others and in developing relationships.

*Yeah, it was very hard … I got really excited meeting other Black kids in high school, you know? And then I realized they were just different also because they grew up in North America. You know? So, they were from the States or they were born in Canada so that wore off really fast. And then they also saw me as weird because you know, I was different to them too …*

(IW08)

For some IWs, the loss of and lack of social support made them question their decision to immigrate to Canada.

*I was like I felt maybe I was like thinking to myself maybe I made the wrong decision moving to Canada. Yeah, because you know I was alone. I didn’t have people to talk to and I didn’t have access to the phone, so it was like it’s something new that happened to me.*

(IW11)

Some IWs who came from collectivist origins found it difficult to change their way of being, because in Canada, the individualistic way of being predominates.

*So I know that that aspect can be difficult for our newcomer women because they’re used to just like being out on the street and chatting with people and um you know, not people running from here and they are keeping to themselves.*

(CSW06)

IW participants reported feeling excluded, and this related to the multiple intersecting social identities, determinants, and structures they needed to navigate. Some were forced to obtain jobs that undermined their knowledge and skill level. Government services and programs prioritized labor market integration, and sometimes, there were limited opportunities that involved social integration. Additionally, IWs experienced exclusion due to issues of inadequate, affordable, and clean housing; lack of financial credibility; limited access to social spaces; and social support in their communities.

### 3.3. Theme: “Paving a Path for Myself”

Experiences during settlement and thereafter were described as a continuum by IWs. This transition reflected how women navigated social and interpersonal spaces and formed connections that made them feel a sense of belonging and empowerment and, in turn, socially included. IW participants described that co-ethnic bonding, engagement in faith-based institutions and multicultural centers or schools, and the workplace made them feel part of the community. Some formed bonds that empowered them to gain self-confidence and to advocate for themself. The following sub-themes of social connections and belonging as well as confidence and empowerment will be discussed.

#### 3.3.1. Sub-Theme: Social Connections and Belonging

Most IWs and CSWs discussed the need for engaging in the new community as being important to feeling included. Connections made with support people encouraged IWs to achieve their goal. Support from co-ethnic community members was particularly important and gave the IWs strength to carry on especially when they had immigrated alone.

*Like I met a friend here, she’s from my home country but she’s been here for a while, and she’s been like a sister and a pillar of strength to me. Like she, you know encourages me and supports me and you know, helps me to, you know, participate … and you know pushes me kind of.*

(IW15)

Sometimes, support gained from one individual made a lasting impact that facilitated cultural transitions, particularly when it came to understanding how participation offered social, economic, and civic benefits. One participant shared that a friend encouraged her to volunteer, which helped her learn about the Canadian culture.

*I think she [friend] was the most impactful. She really, really made a difference because she will drive us everywhere, to Toronto, to learn, to discover things. She really helped us, you know, learn a lot like in general, like the culture and everything right. In Mexico, you never volunteer. Why would you work for free? And she was the one that said, “oh, maybe you should volunteer.” And of course, at the beginning I was like, I’m not working for free, and she’s like “it’s not free. You’re learning skills. They’re paying you with knowledge.”*

(IW13)

Another participant embraced her new culture and created her own sense of belonging through intercultural engagement. She discussed “*pushing* [herself] *to be vulnerable*” (IW13).

*I almost made it a chore where my family had to go celebrate every community event. Thanksgiving, Christmas, Canada Day, and not only go, we had to get the shirt and go with the program … my son is Canadian and I didn’t want to alienate him from his culture because I’m Mexican … they have the right to know the holidays and celebrate it, right? So that helped me a lot because then I pushed myself to talk to people and to ask questions.*

(IW13)

Embracing social connections and intercultural communication were helpful for IWs in navigating cultural transitions, learning about Canadian culture, gaining support to integrate in a new country, and ultimately, made them feel socially included.

#### 3.3.2. Sub-Theme: Confidence and Empowerment

IWs described their experiences of gaining confidence and finding ways to participate in the community and, in turn, were empowered to advocate for themselves and others. Gaining confidence came from social support, language acquisition, having meaningful work, and volunteering. While transitions to a new host society created emotional toil and potentially distress, the IWs had supports that made them feel included and helped them to regain self-confidence through other connections to women who supported them to be empowered to take control of their own lives/social contexts.

… [name of support person] *has played a big part in my life. When I met* [name of support person] *and started relating to her, she’s one of the people that helped me to like gain my confidence, speak up. She told me … if you’re not happy, you have a right to say it. When people say this to you, you have the right to speak up for yourself so I think interacting with her, it really made a difference in my life a lot.*

(IW11)

For some IW participants, the language barrier made it difficult to participate in the community upon arrival, although with language acquisition, intercultural communication increased and so did a sense of being part of the community.

*… I can communicate better. Like, I now think I share more things with my friends, my Canadian friends, like again, more in the community, like we talk about a new place in St. Catharines, or we watch this new TV series so things like that. So now, we can share those things because of the language.*

(IW02)

Most IWs expressed how the ability to communicate with others facilitated social integration and contributed to feelings of comfort. Collectively, improving language ability and meaningful work were influential in building confidence and feeling a sense of purpose in helping others.

*I’m more confident now …, you know, and I feel like I can do anything. I talk more now. I could barely speak, you know, like when there were people around, I’ll just shut down, you know, but now it’s different …, I can. I can do that, and I know I can do it. Even at work I’m so confident, I can give the care that I need to and I actually really, really enjoy my job because of all these people and how they encouraged me.*

(IW15)

Volunteering at community events also helped IWs to build confidence, connect with others in the community, and, as one woman, stated, “*paved a path for myself*” (IW08). The confidence that IWs gained with meaningful support and employment not only provided a sense of fulfillment but also empowered them to disrupt racist behaviors and advocate for themselves and for the rights of others.

*Even at work, I sometimes like go meet people who are racist, I sometimes experience racism, but now I’m not afraid to speak up. Like when I see someone is treating me differently, I can … speak up. I can say I don’t think this is right or I know the protocols. I know if it’s at work, I know who to go and talk to, who to report to. Some people, they can’t. They just keep quiet. They just let people treat them bad and now I’m advocating for other people, even at work,* [for example] *when I see that this person is being treated or not being treated fair because of her skin color or because of where she comes from.*

(IW11)

Along with advocacy, one participant recognized the power in reflecting on past experiences and learning from others to deal with racist behaviors and, in turn, decided to approach the experience positively.

*There’s just so much I’ve been through. Some of the stuff, it just feels like it’s in a dark box for me … like because I really don’t want it to influence how I am to people. I realized because of my bad experiences, especially being called like racial slurs and all that stuff, I realized it was pushing me in a direction of wanting to retaliate … it’s almost like I was also building a whole list of things to be racial about towards White people. So, I was really talking about White people in a negative way, almost like they were all the same. There was a point where I had to just sit back and educate myself … about like, you know, they’re not all like that.*

(IW08)

Another participant (IW07), who did not wish to be audiotaped, described feeling “*ignored*” and “*avoided*” at her educational placement at a childcare center by the parents of the children. She felt that the parents feared her because she was wearing a hijab and they looked at her “*differently*”, which made her feel that they could not trust her. IW07 decided to take the lead and make a greater effort in being kinder to others to gain their trust and acceptance. When women felt accepted by others, this gave them the confidence to be themselves and participate fully.

*That’s where I’ve seen myself participating a lot. I’ve been included. I’m not excluded. In my team, the praise and worship team, I’m the only Black person…I don’t feel it at all when I’m with them. If I compare it to when I was at school … I would feel the difference, that I’m different and I’m not wanted. But when I’m at church. the people are so loving and they make me feel like one of them … I feel free to participate there and I’m confident there because I know I’m part of them.*

(IW11)

Having opportunities to learn the language, work, and connect with others contributed to IW participants’ confidence, sense of empowerment, and ability to develop meaningful social support. Learning from racist behaviors and self-advocacy helped the IWs to feel accepted and participate in the community.

### 3.4. Theme 4: Formal Inclusionary Processes

In most social contexts, formal inclusionary processes represent funded programs that are targeted at newcomers to Canada within a pre-determined time. These programs are important and of significant value to newcomers and often reflect supportive systems to navigate spaces and opportunities in the new country of settlement. In fact, the participants in this study highlighted the importance of community-based settlement and other immigrant/refugee programs that were of value to them during their settlement journey. The availability of culturally diverse and sensitive services and programs were important for them to find their way in the new context. There were many positive experiences portrayed by IW participants of the support or gateway to skill development, language learning, and career opportunities. Additionally, IWs discussed the ability to engage in focused activities, such as cooking classes and excursions to local tourist destinations to learn about and engage with the community. The subsequent sub-themes represent the recommendations that IWs and CSWs believed could be enhanced in formal programs to promote inclusion.

#### 3.4.1. Sub-Theme: Awareness, Access, and Connections

The participants expressed the need for improved access to information support and awareness of available resources and community opportunities. Key recommendations included the following: (1) using digital technologies for information dissemination, community navigation, and to assist in fostering social connections; (2) creating newsletters or information packages of community events and programs to spread awareness; (3) CSWs making an increased physical presence in the immigrant communities to promote available resources and opportunities; (4) making the information resources accessible to immigrants by providing them in multiple languages or using simple pictures to overcome language barriers; and (5) creating local multicultural events and offering cultural inclusion and competency training to raise cultural awareness and strengthen community connections.

#### 3.4.2. Sub-Theme: Women-Centered Programs

Most participants felt that it was important to provide women-centered programs for immigrant adults and youth that assist in empowering them, building confidence, developing friendships, and gaining leadership skills. These programs may provide an opportunity for IWs to gather, feel comfortable and safe to have conversations, share stories, and exchange ideas with other women.

#### 3.4.3. Sub-Theme: Workshops and More Accessible Programs

Workshops that are centered around the creation of activities that reflect IWs’ unique needs, desires, and interests were recommended by most participants. For example, many IWs stated that they bond socially through learning and common interests, such as the environment and food from their cultures. This includes the importance of creating education workshops, tools, and programs designed to support skill development, career building, and learning digital technologies. Moreover, issues related to program accessibility for IWs were described, namely language and transportation barriers and the affordability or timing of programs. Improvements in program accessibility were recommended: for example, creating culturally sensitive programs by offering them in multiple languages or providing virtual options to overcome transportation issues.

#### 3.4.4. Sub-Theme: Learning the Structural Systems and Norms of Canada

IWs need the tools and guidance to help them navigate the structural systems and norms of Canada. Key recommendations included the need to improve translation services to overcome language barriers, specifically improving interpretation services to help newcomers understand Canadian immigration documents or legal paperwork or for when they need to communicate important information with others, such as health-related issues. Furthermore, a strategy recommended by the CSWs included intercultural mentorship programs where Canadian volunteers are partnered with newcomer families to assist them in learning Canadian social, health, and economic systems and to acculturate.

### 3.5. Theme 5: Informal Inclusionary Processes

Given the stressors of settlement, having informal programs available and accessible may give IWs the cushion they need to land in the settlement country so that these transitions are not only easier but also so that they feel accepted, welcomed, and included in their new social space and context. Informal inclusionary processes reflect small-scale programs led by the community that are sometimes taken for granted and often are not valued because they do not demonstrate rigorous evaluation of their impact [39,40,41]. As well, they are often not sustainable, particularly if they are not funded consistently. Activities are seen as informal because they do not address a specific area of settlement, such as employment or language learning, but rather they attend to the socialization and engagement with and across communities. In this study, the participants believed that informal mechanisms of recognition, socialization, and acculturation were relatively important.

#### 3.5.1. Sub-Theme: Safe and Inclusive Spaces That Are Open and Welcoming to Difference

IWs felt it was important that they have a space where they felt comfortable opening up and connecting with one another and being empowered because it allowed them to embrace their differences. The CSWs believed these spaces should not only be safe but also free of stress and judgement in order for them to be inviting to immigrant and refugee women and to allow for the celebration of different cultures. They also believed that these spaces should foster happiness and enjoyment, especially given the challenges the women faced.

#### 3.5.2. Sub-Theme: Capitalizing on Women’s Strengths

The CSWs highlighted the positive aspects of women migrating to Canada and felt that there should be opportunities for them to showcase their knowledge, skills, and experiences by sharing with others. The purpose of sharing experiences in informal spaces was to give other women hope for the future given their special skill sets. IWs appreciated the opportunity to offer and receive help from other women in the community in times of need.

#### 3.5.3. Sub-Theme: Creative and Supportive Activities

Overall, the participants felt that programs should encourage participation in creative activities as a means to develop support and new friendships. The CSWs shared how their programs used creative activities to bring newcomers together and learn from one another. In addition to learning a new recipe or skill, the CSWs mentioned that these activities helped with confidence building. Through a specific activity (i.e., knitting newborn hats), there was a common goal that allowed them to contribute in a meaningful way to their new community.

## 4. Discussion

The findings from this study contribute depth of understanding of the intersectionality of social identities and the interplay of social determinants and structures that create challenges for IWs during the transitionary period of initial settlement but also in the long term for some women, particularly those who did not achieve social or psychological adaptation to the new context. A discussion of the findings in tandem with the existing literature will be presented with a focus on the themes, including recommendations for strengthening systems to create more inclusive spaces and women-centered programs. A brief discussion of how this knowledge advances health equity in programs and future research will also be presented.

Immigrant women put aside their careers whilst honoring their cultural norms and practices, and for some, this delayed their entry into social, cultural, economic, and civic domains within the new host society. However, this was not seen as a hindrance or disadvantage but rather a requirement because of the expectations placed on immigrant women to be the primary caregivers for children and hold the responsibilities of caring for the family and household. Prioritizing the husband’s career is not an uncommon finding, irrespective of ethnocultural background. In one qualitative study, Turkish women settled in the USA prioritized their husbands’ careers because of cultural norms and responsibilities, which placed the onus on them for the reproductive labor in the family [41].

If IWs are confined to their home for an extended period of time, this leads to individual concerns about capabilities related to delayed entry into the labor market, as found in our study. This was an issue that was also raised by Chinese immigrant mothers settling in Toronto, Canada, where they experienced “boredom” and “self-enclosure”, which did not contribute to their well-being, and they worried about delayed integration over time and being able to adapt or manage the context or changes in the employment sector [42]. In the settlement context, the delay in entry into the labor market intersects with other domains, such as social, cultural, or civic engagement. This reflects a delay in accomplishing language learning, establishing new social networks, developing relationships, and volunteering. Often, immigration status dictates what women are eligible to access in the new country of settlement, and while there may be programs that could assist them to participate, they may be ineligible. As evident in our study, IWs who accessed settlement services did so but experienced challenges related to programs not accommodating to diverse needs, such as language. Often, settlement programs cater to the “average” immigrant or refugee, and, in turn, programs are designed without considering the diverse trajectories along the life spans of individuals and unique social identities [40]. Immigrant women in the USA reported facing financial troubles due to exclusion from “restrictive policies” and the resources and services required to support their intersecting social identities, which further perpetuated the gendered nature of exclusion [43]. Not only are there challenges for some accessing settlement services, which was evident in our study, but this often delays entry into the labor market. Delayed entry may also reflect a lack of guidance from settlement services, as was found in a qualitative study conducted in Finland; an immigrant woman was told to return for integration support services when her child was older, and when she did, she was no longer eligible for these services [44]. Across OECD countries, settlement programs are set up to support newcomers, including refugees over a short period of time; however, the reality is that IWs may not be able to make use of these services within the first three years of settlement and, thus, are not supported when they are ready. As noted in one report, integration into the labor market for refugee women is delayed and may take 10–15 years compared to male refugee counterparts [45]. Entering the labor market opens up opportunities to participate in other social, cultural, or civic domains.

Even after women were at a point where they were able to join the labor market in our study, the challenges included accepting employment that did not align with their education or professional career because of devaluing, or they needed to juggle “survival jobs”, which intersected with role responsibilities. Juggling work and role responsibilities was also experienced by Chinese immigrant mothers who were discouraged from seeking out employment related to prior professional careers due to licensing or, in the case of other women, not having the time to participate in language training [42]. Devaluing education, qualifications, and experience in their professional field was a form of exclusion that was also identified in a study among Asian women settling in Australia [46].

As uncovered in our study, the more challenges women faced upon immigration, the greater the exclusion. Women have discussed many incidences of othering, racism, and discrimination, which compounded exclusion. Similar to the women in our study, immigrant women in the USA shared experiences of intercultural contact in public spaces, such as workplace, schools, and healthcare settings, that resulted in xenophobic and discriminatory comments and “attacks” about physical appearance, clothes, and language differences [43]. Immigrant women in another study also reported stigma because of wearing a hijab related to fear, and assumptions of culture, religion, and perceptions of Muslims fueled the stigma [47]. In Canada, the Muslim women (born outside and in Canada) interviewed in one study believed that fear was precipitated by negative public media about Muslims, and this translated into misconceptions of them and what they represented [48]. Given the othering in social spaces, it is not surprising that immigrant and refugee women look to co-ethnic communities and women for support. The choice of acculturational strategy used may be influenced by acceptance in the host country; for example, immigrant and refugee women may adopt the marginalization strategy [49]. In this case, being racialized and stigmatized reduces the desire to engage with the host society, and thus, women choose to stay within their co-ethnic communities because it is safe. Although engagement with co-ethnic communities may be best initially, moving towards more intercultural connections would facilitate greater integration and inclusion within a new society [49].

In our study, most IWs mentioned the connections they made with people that supported them to gain knowledge and skills that empowered them in navigating social spaces in Canada. Co-ethnic support was vital to women upon settlement in our study, as was the different levels of support. Support made IWs feel connected, a finding also reported in a study with newcomer women to Canada, where co-ethnic communities provided instrumental, informational, and, sometimes, emotional support [50]. Also, peer support was reciprocated in that it inspired newcomer women who received co-ethnic community support to volunteer in settlement/multicultural agencies to provide support to other women [50]. This made them feel valued and increased their confidence and self-esteem.

Gaining confidence came from language acquisition, having meaningful work, volunteering, and social support, which, collectively, empowered IWs to achieve their goals in our study. Likewise, this was found in one qualitative study that highlighted language acquisition as an endpoint for one participant to obtain a better career; she stated that to do this, she capitalized on resources, was determined, and learned the language [51]. In our study, obtaining fulfillment in one’s career or employment enabled IWs to decide what was best for them. This finding resonated with another study, where immigrant women adapted to their circumstances in relation to their career aspirations and delay in being able to obtain full-time employment [41]. The women in Elitok and colleagues’ [41] study “re-defined their ambitions” to reimagine the benefits that migration offered their children and looked at what was important to them to be happy in life. Similar to Rashid and Gregory’s [51] study, despite the struggles experienced due to migration, highly educated immigrant women to Canada shifted their expectations of career aspirations and made the most of their experiences so that they found meaning and purpose and, in turn, happiness.

In our study, social exclusion as a process and subsequent step that led to feelings of inclusion was elucidated by IWs in their stories of strength. While the initial phases of settlement are fraught with challenges in regard to finding their way in social and physical environments, struggles and resilience to form connections eventually led to empowerment and belonging for most IWs in our study. Community resources and opportunities helped them to feel empowered. This was also evident in other studies with acceptance of the new life and opportunities in the host country, along with strong conviction and work ethic, courage, and determination, which collectively assisted immigrant women to gain a sense of belonging in the USA [52]. Embracing the opportunities and resources helped them to adjust to their new life. This process of adjustment relates somewhat to the theory of well-being during cultural transition that was generated from a study in the USA among Sudanese women, where they progressed through separation, liminality, and integration [13]. Unlike voluntary settlement, refugee women left their home, family, and culture due to political persecution and needed to adjust to new cultures, norms, and spaces in the USA. Liminality reflected a state of vulnerability, as women did not feel like they belonged in their Sudanese culture nor to the American culture claiming, they were “in-between” [13]. With this transition comes confusion, disorientation, and uncertainty but also a sense of newness and discovery, which may reflect a positive change. How long a woman remains in the liminality phase will depend on the individual ability to learn the new language, establish social networks, and secure employment. The transformation of one’s identity that facilitated integration reflected internal factors, such as values and beliefs of the new culture and integration into one’s own personal identity, and external factors, such as becoming a productive member in one’s community through language skill development, seeking employment, and civic engagement [13]. As women navigated their new culture, they also became more independent and confident, resulting in changes within themselves. Together with this renewed identity, women’s roles also changed in their family and social networks.

In our study, community-based and settlement programs were perceived to be of value if women from diverse communities were fortunate to have the awareness to access these services and/or opportunities. The social dynamics of navigating spaces are influenced by intersecting social determinants of health. In our study, spaces where the women did not feel included were centered around a lack of exposure to diversity in social spaces or an inability to participate due to difficulties navigating services or social events. However, as with any programs for settlement and integration, there were areas of improvement that focused on the intersectionality of IWs’ experiences. While all settlement services embrace equity, diversity, and inclusion, there are aspects of these spaces that may not always accommodate to the gendered nature of immigrant and refugee women’s lifeworld, as seen in our study. Acknowledging that the intersections of the social determinants of health create different levels of disadvantage for women is essential [4]. Accommodating these spaces by attending to cultural- and gender-based needs may reduce social exclusion and risks to health and well-being. The recommendations provided by the women in our study reinforced the need to increase the awareness of services and women-centered programs and gain cultural knowledge of the systems and norms in Canada. These findings are in line with other research, where it was identified that the existing programs need to be reconceptualized to consider the unique needs of diverse women upon settlement and beyond [40].

In particular, women may not immediately benefit from labor market integration programs, but rather they may benefit from programs that build social capital (i.e., social support network) and incite a sense of belonging in the new setting [40]. Acknowledging the delay into the labor market entails working collaboratively with community/civil society organizations that will support and coordinate informal services for women over the long term [40]. Settlement and integration programs need to be rethought; immigrants/refugees have diverse social identities and are at different stages of the life course; therefore, their work and life goals will differ. There is a call for governments to value and invest in social programs, as these are interconnected with economic programs. An investment by governments in community-based informal initiatives would involve intersectoral collaboration with the goal of filling the gap in current programs. The overall benefits are not only to individuals but their families and society as well [40].

While informal programs are less valued and rely on small pockets of funds that are not sustainable, they reflect the work that is needed to promote social inclusion, especially for migrant women. In our study, IWs reported that they felt included if they were in safe and inclusive spaces that made them feel welcomed; the programs valued women’s strengths; and more creative activities were planned to engage them. These findings align with a scoping study focused on social inclusion interventions that highlighted the use of peer-led strategies incorporating aspects of support, including art-based interventions capitalizing on pre-existing skills from origin countries [53].

Future research using longitudinal studies is required for evaluating women-centered informal programs that aim to tend to social, cultural, and civic participation while also enhancing the social networks required for economic and social inclusion. Collaborative relationships with organizations that offer formal programs will be vital in supporting evaluation, as they have the knowledge and skills. Additionally, working closely with universities can leverage research and evaluation support with a memorandum of understanding, as is the case with the collaborative partnership that was established for this project.

### Limitations

One limitation in this study was the longer time needed for recruitment during the pandemic, as we were slow in reaching IWs mainly via email or community-organization website posts and had to repost our messaging numerous times. We learned that IWs preferred to use WhatsApp for communication and were less inclined to follow social media posts. Our community partners helped with spreading the word by disseminating our poster in different spaces. Also, some IWs may not have felt comfortable with being interviewed virtually or lacked computer access, even though we did offer telephone interviews. Therefore, our sample may not have captured the voices of IWs who experienced great levels of social exclusion, including not being connected to settlement services or other community spaces. If we had been able to recruit on the ground and in person, we may have been more successful in gaining access and familiarity with the communities, so that we could reach IWs in person. Additionally, we were unable to recruit IWs who had resided in Canada for less than five years (newcomers) and 10–15 years (long-term residents), which are two groups that reflect diverse experiences and, perhaps, different levels of exclusion. We know that their experiences may have differed based on their reasons for migrating, origin countries, family composition, and sense of belonging, and thus, our findings may not resonate with these IWs. Lastly, we did not use interpreters for interviews, even though they were available to us from the multicultural center. All participants who volunteered for our study were able to converse in English, which is a limitation, as their experiences likely differ from those who experience language barriers upon settlement. Women with language barriers and other intersecting social determinants, such as the social environment, serve to exclude IWs, as they may not be unable to participate in social, economic, or civic activities. Thus, our findings should be interpreted with caution, as our participants do not represent IWs who may experience greater disadvantage and exclusion related to language barriers and, in turn, have different stories to share.

## 5. Conclusions

This study contributes to knowledge of social exclusion experiences and social inclusion strategies that may be used among immigrant and refugee women within the Canadian context or beyond. Our programs are somewhat limited to settlement services that focus on economic integration, including language learning, that caters to the average immigrant and refugee population over a short period of time. Consequently, this fails to meet the needs of IWs who delay their entry to participate in social, economic, cultural, and civic activities in their new communities. The recommendations made to improve formal programs for settlement and integration and the integration of informal programs are of significance, as they aim to meet the needs of immigrant and refugee women, a population that continues to experience social exclusion in many contexts. The incorporation of an intersectionality framework is critical in formal program modifications or the development of informal programs.

The practical implication of these findings will be valuable to inform the next steps in our sociocultural community to improve social inclusion for newcomer and long-term resident immigrant and refugee women. It is hoped that a collective response among community organizations and members will facilitate the development of women-centered programs that serve to prevent social exclusion and improve their health and well-being. Future research is necessary to evaluate the informal programs developed specifically for immigrant and refugee women. Until we consider the gaps and incorporate more sustainable informal programs in the social spaces of our communities to meet the needs of immigrant and refugee women, we will not be able to actualize health equity.

## Figures and Tables

**Table 1:** Demographic characteristics of IWs *.

IWs Demographics	Percentage (%)
Number of participants N = 10 Age (mean = 41.5 years), years	
20–29	20
30–39	10
40–49	50
50–59	0
60+	10
Did not disclose	10
Racial or ethnic identity	
African Arab	10
Black	40
Chinese	10
Latin American	20
White/Caucasian	10
Did not disclose	10
Average residency length in Canada (mean = 14.8 years), years	
1–10	40
11–20	30
21–30	10
31–40	10
40+	0
Did not disclose	10
Completed education level	
Less than high school	0
Completed high school	30
Completed trade, certificate, or diploma	40
University degree (Bachelor’s)	0
Post-graduate degree	20
Did not disclose	10
Employment status	
Employed outside the home, full time	60
Employment outside the home, part time	20
Retired	10
Did not disclose	10
Living situation	
Lives with partner/spouse and children	10
Lives with partner/spouse only	30
Lives with parents	20
Lives alone	30
Did not disclose	10
Marital status	
Married	30
Divorced/separated	30
Never married	20
Did not disclose	20

* Please note that two participants did not complete all items in the sociodemographic form.

**Table 2:** Demographic characteristics of CSWs.

CSW	Population Served	Programs Offered	Outreach Methods	Organization Partnerships
CW01	Newcomers and the public	Environmental workshops, community garden, and events and festivals	Social media, in-person presentations, and word of mouth	School boards, multicultural centers, college, and municipality
CSW02	Family and children	Family and child support services	Telephone	Children centers and community day cares
CSW03	Newcomers	Recreational activities and mentoring	Virtual outreach	Food/nutrition agencies and heritage council
CSW06	Newcomers	Education and training, language, housing, financial assistance, and events and festivals	Social media, virtual outreach, and word of mouth	Multicultural centers, municipality, provincial agencies, school boards, financial institutions, community healthcare providers, faith-based charitable organizations, and public library
CSW07	Newcomers	Language, housing, employment assistance, family and child support services, and recreational activities	Referred	Multicultural centers, municipality, college, and faith-based charitable organization
CSW08	Immigrants and newcomers	Employment and school assistance	Not specified	Multicultural centers, heritage council, school boards, provincial agencies, and municipality
CSW09	Newcomers	Recreational activities and volunteering	Posters, virtual outreach, word of mouth, and texting apps	Recreational agencies and food/nutrition agencies
CSW10	Immigrants and newcomers	Recreational activities and digital literacy	In-person presentations and community visits, virtual outreach, and word of mouth	Provincial agencies, recreational agencies, multicultural centers, and municipality
CSW11	Immigrants and newcomers	Employment and training assistance	Not specified	Local employers
CSW12	Immigrants, newcomers, and refugees	Employment assistance	Referred	Multicultural centers and community shelters/housing services
CSW13	Immigrants, newcomers, and refugees	Employment assistance and skills building	Social media, virtual outreach, and word of mouth	Financial institutions and employment centers
CSW14	Immigrants and newcomers	Family and child support services and language assistance	Not specified	Regional, university, and recreational agencies
CSW17	Newcomers	Youth services, life skills, education and employment assistance, and mental health services	National border-crossing locations, public institutions, and word of mouth	Heritage council, multicultural centers, recreational agencies, municipality, health centers, school boards, faith-based institutions, national agencies, and provincial agencies
CSW18	Refugees	Housing and transportation assistance	Referred	Multicultural centers, employment centers, school board, and faith-based committee

## Data Availability

This research did not use a publicly available data set.
